# Parameterization of the Age-Dependent Whole Brain Apparent Diffusion Coefficient Histogram

**DOI:** 10.1155/2015/373716

**Published:** 2015-11-02

**Authors:** Uwe Klose, Marion Batra, Thomas Nägele

**Affiliations:** Department of Diagnostic and Interventional Neuroradiology, University Hospital Tübingen, Tübingen, Germany

## Abstract

*Purpose*. The distribution of apparent diffusion coefficient (ADC) values in the brain can be used to characterize age effects and pathological changes of the brain tissue. The aim of this study was the parameterization of the whole brain ADC histogram by an advanced model with influence of age considered. *Methods*. Whole brain ADC histograms were calculated for all data and for seven age groups between 10 and 80 years. Modeling of the histograms was performed for two parts of the histogram separately: the brain tissue part was modeled by two Gaussian curves, while the remaining part was fitted by the sum of a Gaussian curve, a biexponential decay, and a straight line. *Results*. A consistent fitting of the histograms of all age groups was possible with the proposed model. *Conclusions*. This study confirms the strong dependence of the whole brain ADC histograms on the age of the examined subjects. The proposed model can be used to characterize changes of the whole brain ADC histogram in certain diseases under consideration of age effects.

## 1. Introduction

Diffusion-weighted imaging provides additional image contrast in MR imaging of the brain and has become an important part of clinical MR diagnostics. Data evaluation is usually performed directly in the diffusion-weighted images or in calculated apparent diffusion coefficient (ADC) maps. In addition to the analysis of images and maps, the distribution of ADC values in selected region of the brain can be used to characterize the examined tissue. This was shown, for example, for stroke lesions (lesions with subsequent hemorrhagic transformation could be identified by the ADC histogram) [1], for multiple sclerosis patients [2], for low-grade and high-grade gliomas [3, 4], and for epilepsy [5]. In addition, histograms from the whole brain can be used to characterize global changes of the brain tissue. This possibility was introduced by Mascalchi et al. [6, 7] and also by Molko et al. [8]. Mascalchi et al. found a correlation of the median ADC value with a disease score in patients with leukoaraiosis, while Molko et al. examined patients with cerebral autosomal dominant arteriopathy with subcortical infarcts and leukoencephalopathy (CADASIL) and found that the histogram parameters mean ADC value, peak height, and peak locations were significantly correlated with both the Minimental State Examination score and Rankin Scale score in the patient group. These parameters were also examined in other studies [9–14]. In more sophisticated studies, further parameters such as 25th and 75th percentile, kurtosis and skewness were evaluated [5, 15]. All groups excluded pixel outside the skull; some groups also excluded pixels containing cerebrospinal fluid (CSF) and extracerebral tissue based on a manual [8, 10] or automated segmentation [11]; and others excluded CSF by an ADC threshold [7, 12]. Only few groups performed a modelling of the ADC histogram: Pope et al. suggested a two-mixture normal distribution [16], and Dyke et al. and Zhang et al. used a model consisting of two normal distributions for the brain tissue and CSF and an additional distribution for partial volume of the brain tissue and CSF [17, 18]. All these groups did not include an age dependence of the ADC histogram in their model estimations. Recent studies showed a strong influence of age on the shape of the whole brain histogram [19, 20]. In particular, the relative content of cerebrospinal fluid in the brain is increasing with age and this has an effect on the ADC histogram. The examination in this study was performed with a large number of datasets. This allowed the calculation of highly reliable average histograms for different age classes.

The aim of this study was the development of a model for the fitting of the ADC histogram. The contribution of brain tissue (gray and white matter) is responsible for the left part of the histogram. In a first step, a model for the large maximum of the ADC histogram should be developed and, in a second step, a model for the right part of the ADC histogram was searched. In both cases, the influence of age was considered.

Such models of the description of the ADC histogram in normal subjects should be helpful for the description of changes of the ADC histogram in patients with certain pathologies.

## 2. Methods

### 2.1. Subjects and Data Acquisition

All examinations were performed with a conventional 3T MR whole-body scanner Skyra (Siemens Erlangen, Germany) equipped with a 20-channel head coil as part of the standard routine examination of patients with neuroradiological report requests from several departments of the University Hospital of Tübingen. Informed consent was obtained after the nature of the procedure had been fully explained. The local Ethics Committee approved of this retrospective study.

Data were acquired between August 2012 and July 2014. In all cases, the diffusion-weighted readout-segmented echo planar imaging (rs-EPI) sequence was applied with identical measurement parameters: repetition time (TR) 6.3 s, echo time (TE) 73 ms, b-values 0 and 1000 mm/s^2^, matrix 224*∗*224, FOV 230 mm, slice thickness 4 mm, slice gap 0.8 mm, 30 slices, number of segments of the segmented EPI-sequence 5, and diffusion gradient scheme: three-scan trace. All measurements were performed in axial orientation parallel to the line through anterior commissure and posterior commissure (AC-PC line). All DWI data were carefully examined and data with pathological changes or with artifacts were excluded from evaluation. The exclusion of datasets was based on the decision of two neuroradiologists with more than 10 years of experience in the MR examination of patients.

The total number of examinations was 1327 patient examinations, and after the data exclusion 891 remained (448 females, 443 males). From all patient data, seven age classes were built: 10–20 years (group 1, *n* = 75, 28 females, 47 males), 20–30 years (group 2, *n* = 104, 63 females, 41 males), 30–40 years (group 3, *n* = 103, 61 females, 42 males), 40–50 years (group 4, *n* = 155, 86 females, 69 males), 50–60 years (group 5, *n* = 161, 78 females, 83 males), 60–70 years (group 6, *n* = 134, 51 females, 83 males), and 70–80 years (group 7, *n* = 113, 62 females, 51 males).

The age was calculated by the difference between date of measurement and date of birth and used as a decimal number. Patients of the first class had a calculated age equal to or larger than 10.00 years and lower than 20.00 years. The separation into the six other age groups was correspondingly calculated. Patients with age lower than 10 and those older than 80 years were not included in this evaluation, since the number of these patients' groups was too small and their ages were not equally distributed within these groups.

### 2.2. Calculation of ADC Histograms

For each of the age groups, average ADC histograms were calculated for all patients and for female and male separately and a fitting procedure was performed (described in the following). In addition to the age-dependent evaluation of the whole group of examinations, three subgroups of datasets were built and all fitting procedures were applied to each of the subgroups. The results from these three additional evaluations allow an estimation of the reliability of the obtained results of the whole group evaluation.

Each subset was built from patient examinations from a certain range of measurement dates. The number of datasets within the subgroups was 298 (147 females, 151 males), 298 (141 females, 157 male), and 295 (160 females, 135 males). The mean age of the whole group was 47.7 years; the mean age of the three subgroups was 47.5, 47.7, and 48.0 years.

**Figure 1:**
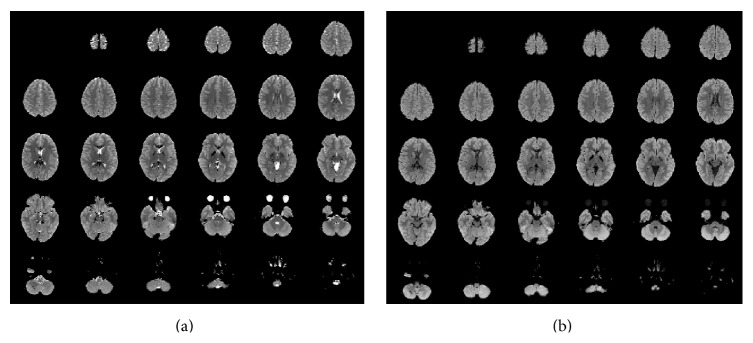
Example of acquired data from one patient: b0-images (a) and dw-images (b).

All fitting procedures were also performed for these subsets to have a possibility to estimate the stability of the fitting results. Typical images with the applied sequence are shown in Figure 1. All 30 slices of the b0-images (Figure 1(a)) and the dw-images (Figure 1(b)) are shown.

Scaling of the acquired data was not consistent. Therefore, a histogram of values in the dw-images was evaluated for each patient on the basis of all 30 slices. The maximum of the smoothed histogram was estimated. The signal intensities of the acquired b0 and dw data were divided by a scale factor derived as the quotient of this median intensity and an arbitrarily chosen value of 250.

Noise pixels and pixels from the skull were excluded by applying a combined threshold derived from b0- and dw-images: pixels with a dw-value lower than 130 and b0-value lower than 600 were selected for exclusion. This procedure avoids the exclusion of pixels containing CSF with low dw-values and allows the exclusion of lipid pixels from the skull.

For the remaining pixels, the ADC value was calculated and histograms of all ADC values were evaluated for each patient. The histograms were normalized to the number of pixels exceeding the noise threshold. This normalization compensates for different head sizes.

### 2.3. Models for the Fitting of the ADC Histograms

From an average ADC histogram over all groups, the position and the height of the peak were evaluated. Next, the positions of the ADC values at the half-peak height (full width at half maximum, FWHM) below and above the peak position were evaluated and named as ADC_50%_below and ADC_50%_above. The position of the ADC_50%_above value was used to split the mean histograms of all seven groups in two parts, hist1 and hist2, which were separately fitted to model functions. The first part of the histogram was approximated by two models: a single Gaussian curve and a combination of two Gaussian curves:

(1) Model  1:  hist1=A∗1σ2∗π∗exp⁡−12ADC−μσ2,Model  2:  hist1=hist1a+hist1bhist1a=A1∗1σ12∗π∗exp⁡−12ADC−μ2σ22hist1b=A2∗1σ22∗π∗exp⁡−12ADC−μ1σ12.

The second part of the histograms, hist2, showed a decreasing behavior and was therefore approximated by a two-exponential decay curve (starting at the first point of hist2) and a decreasing straight line. In addition, the contribution of the CSF spaces was approximated by a Gaussian curve:

(2) hist2=hist2a+hist2b+hist2c+hist2dhist2a=A3∗exp⁡−ADC−ADC_50%k3hist2b=A4∗exp⁡−ADC−ADC_50%k4hist2c=A5−m5∗ADC−ADC_50%hist2d=A6∗1σ62∗π∗exp⁡−12ADC−μ6σ62.

The fitting of the first and second part of the histogram was performed for all seven age classes. The similarity between the respective part of the histogram and the fitted curve was described by the root mean square deviation (rmsd). In addition, the relative rmsd (rmsd divided by the mean value of the respective part of the histogram) was calculated.

Calculations were performed by a computer program using MATLAB (MathWorks, Natick MA, USA) written by one of the authors.

## 3. Results

**Figure 2:**
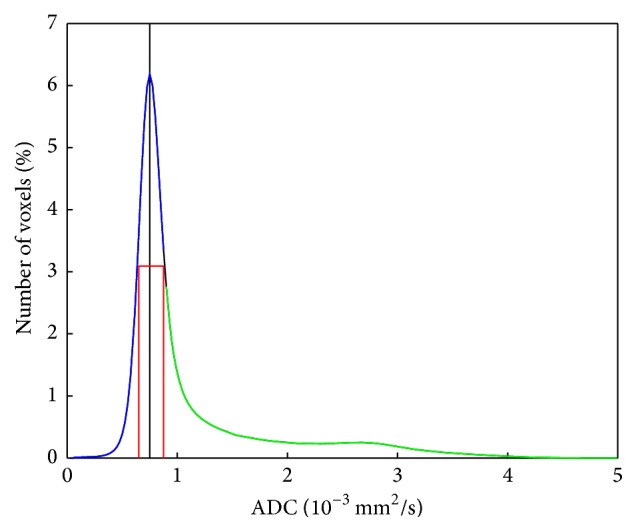
Histogram of ADC values from all patients. The position of the peak is marked by a black line; the position of the ADC values where the FWHM is reached is marked by red lines (ADC_50%_below and ADC_50%_above). The histogram was split into a first part (lower values than ADC_50%_above, blue) and a second part (larger values than ADC_50%_above, green). The value of ADC_50%_above lies between two data points; therefore the part of the histogram between the highest data point of the first point and the lowest part of the second point is shown in black.

The calculated mean ADC histogram over all seven groups is shown in Figure 2.

The peak was at 0.75 · 10^−3^ mm^2^/s and the ADC_50%_above value at half-peak height was at 0.875 · 10^−3^ mm^2^/s. This was the value where the mean ADC histograms were split.

**Figure 3:**
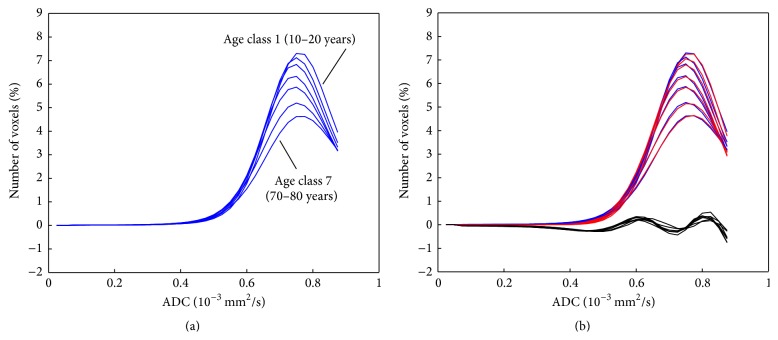
First part of the histograms of all seven age groups (a) in blue and with superposed fitted curve (based on model 1) in red (b). In addition, the differences between histograms and fitted curves are shown in black, magnified by a factor of 3. The histograms of the seven age classes follow a continuous order.

The first part of the mean histograms for the seven age classes is shown in Figure 3.

The results of the fitting of the first part of the histogram using model 1 and the deviations are shown in Supplementary Table 1 (Supplementary Material available online at http://dx.doi.org/10.1155/2015/373716). In Figure 3(b), the fitted curves for all seven classes due to model 1 are overlaid in red. The amplitude *A*_1_ of the Gaussian curves is continuously decreasing with the mean age of the seven classes. The fitting procedure was repeated for males only and females only in the seven age classes. The deviation of evaluated histograms and fitted curves are shown in Figure 3(b), in black. These difference curves show a unique behavior for all seven age classes (the maxima and minima of the difference curve are at the same position for all seven age classes). This shows that the used model 1 is incomplete.

**Figure 4:**
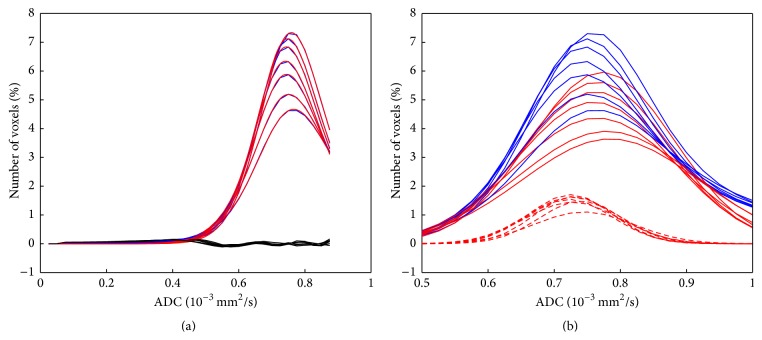
First part of the histograms of all seven age groups in blue and with superposed fitted curve (based on model 2) in red (a). The differences between histograms and fitted curves are shown in black, magnified by a factor of 3. The two Gaussian curves of the fit are separately shown in (b). The blue curves in (b) are again the calculated histograms.

**Figure 5:**
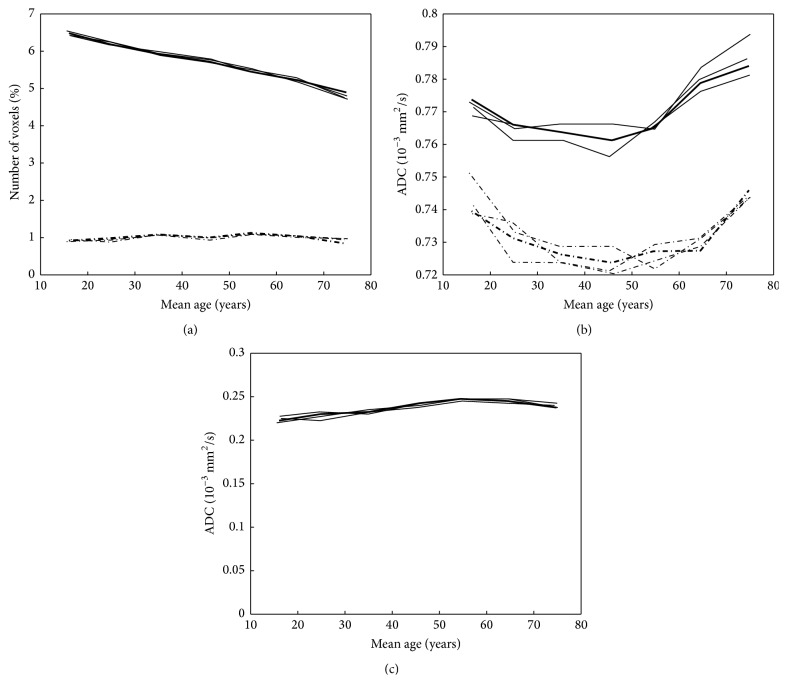
Fitted parameters of the first part of the histogram (based on model 2) for the different age groups: amplitudes of both Gaussian curves (a) and peak positions of both Gaussian curves (b) and of the width (FWHM) of the fitted curve (c). In all cases, the parameters are shown for all selected patients (bold line) and for the results of the three subsets of patients.

The results of the fitting of the first part of the histogram using model 2 and the deviations are shown in Supplementary Table 2. The histograms and the fitted curves based on model 2 are shown in Figure 4(a). The difference curves between the histograms of the seven groups and the fitted curves in Figure 4(a) have all a much smaller maximum than those in Figure 3(b) and they do not show a systematic shape as the difference curves in Figure 3(b). The two Gaussian curves that were fitted to the histograms are shown in Figure 4(b). In all seven age classes, a larger Gaussian curve with a higher mean ADC value and a smaller one with a lower mean ADC value were obtained. The obtained values for the fit parameters are shown in Figure 5. In addition to the results from all patients (shown as bold lines), the results from the seven age classes from the three subsets of patients are shown.

The obtained amplitudes *A*_1_ and *A*_2_ are shown in Figure 5(a). The mean positions *μ*_1 _and *μ*_1 _for the seven age classes are shown in Figure 5(b), and the widths of the fits calculated from the sum of both Gaussian curves are given in Figure 5(c).

**Figure 6:**
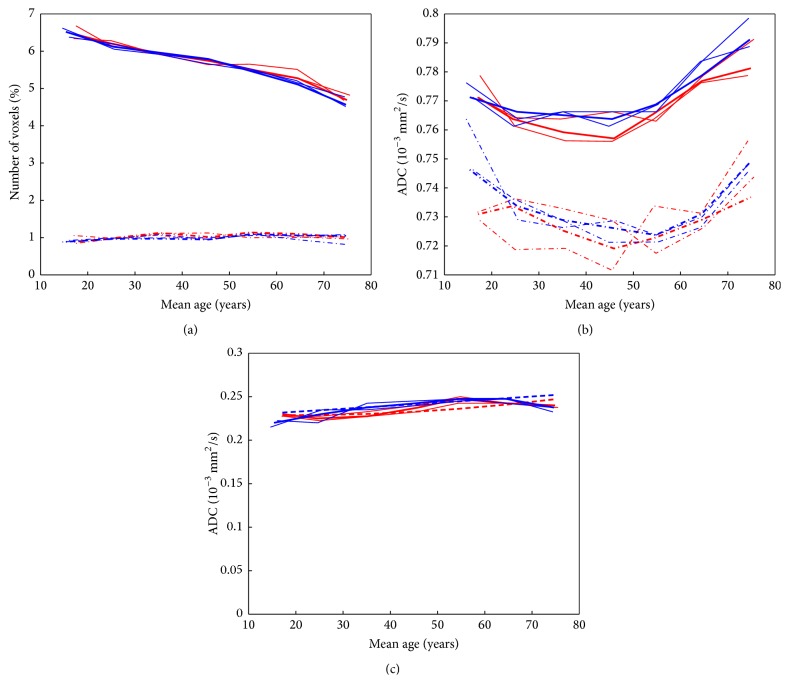
Fitted parameters of the first part of the histogram (based on model 2) for the different age groups: amplitudes of both Gaussian curves (a) and peak positions of both Gaussian curves (b) and of the width (FWHM) of the fitted curve (c). In all cases, the parameters are shown for female (red) and male (blue).

Figure 6 shows the results for the separate evaluation for females and males again for all patients (bold lines) and results from the three subsets of patients. The only clear difference is a larger peak ADC position of men in the age range from 30 to 50 years.

**Figure 7:**
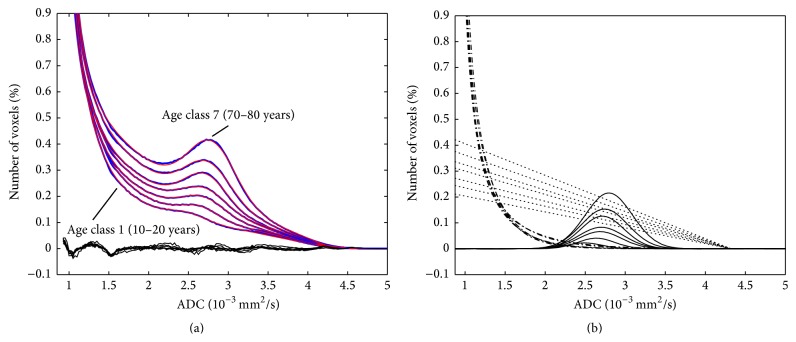
Second part of the histograms of all seven age classes in blue and with superposed fitted curve in red (a). The differences between histograms and fitted curves are shown in black, magnified by a factor of 3. The biexponential decays, the Gaussian curves for the CSF component, and the straight lines are separately shown in (b). The histograms of the seven age classes follow a continuous order.

**Figure 8:**
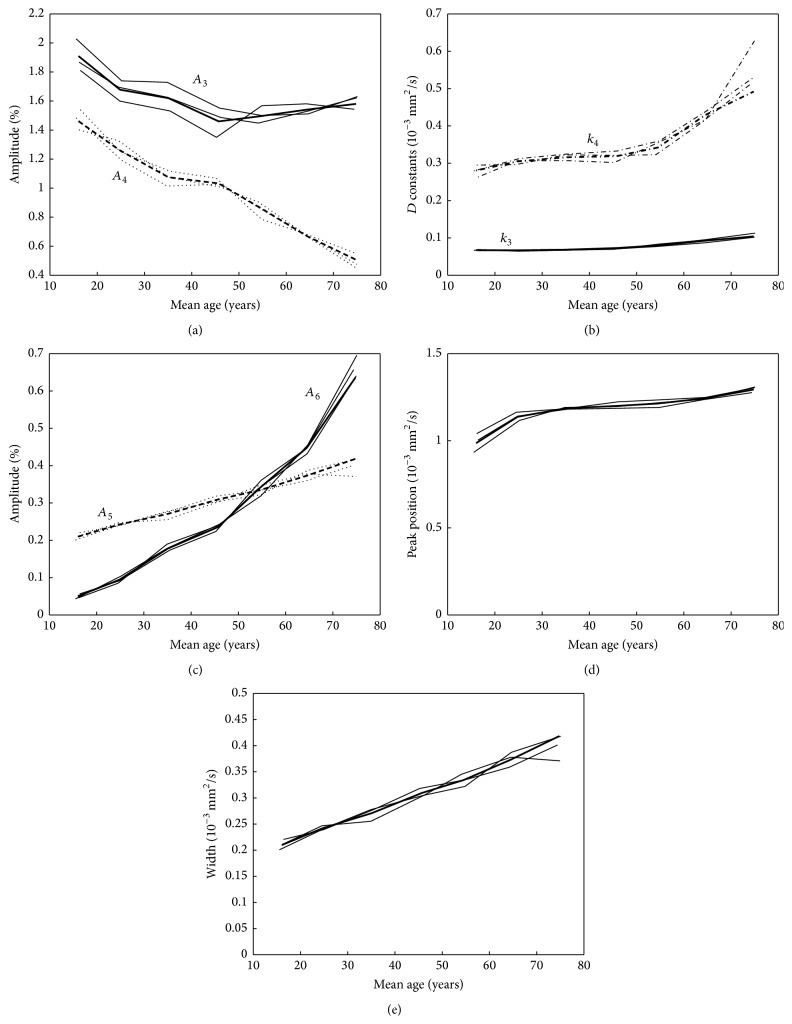
Fitted parameters of the second part of the histogram for the different age classes: amplitudes *A*_3_ (-) and *A*_4_ (- -) of the exponential decay curves (a), the diffusion constants *k*_3_ and *k*_4_ of the exponential decays (b), the amplitude *A*_6_ of the Gaussian curve for the CSF component (-) and the value *A*_5_ of the straight line at the position 0.875 · 10^−3^ mm^2^/s (- -) (c), the peak position *μ*_6_ (d) and the width (FWHM) (e) of the Gaussian curve for the CSF component. In all cases, the parameters are shown for all selected patients (bold line) and for the results of the three subsets of patients.

**Figure 9:**
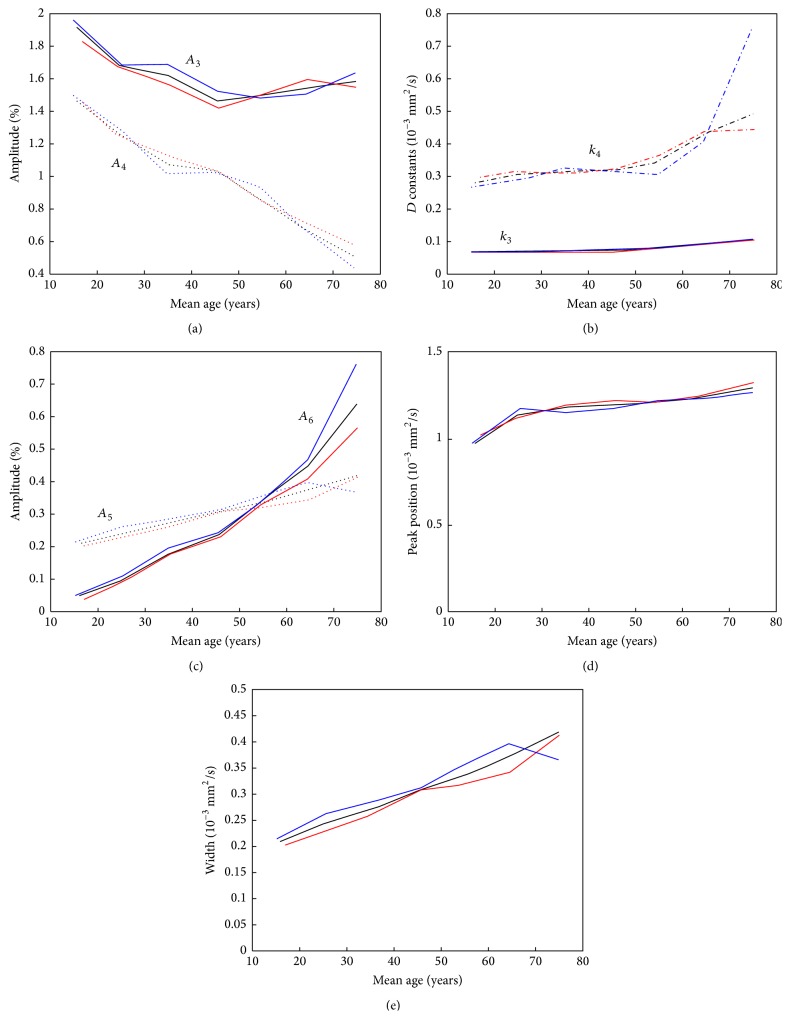
Fitted parameters of the second part of the histogram for the different age classes: amplitudes *A*_3_ (-) and *A*_4_ (- -) of the exponential decay curves (a), the diffusion constants *k*_3_ and *k*_4_ of the exponential decays (b), the amplitude *A*_6_ of the Gaussian curve for the CSF component (-) and the value *A*_5_ of the straight line at the position 0.875 · 10^−3^ mm^2^/s (- -) (c), the peak position *μ*_6_ (d) and the width (FWHM) (e) of the Gaussian curve for the CSF component. In all cases, the parameters are shown for women (red), men (blue), and all patients (black).

**Figure 10:**
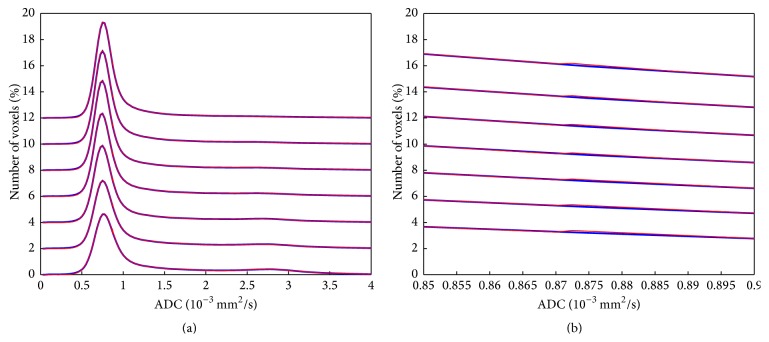
Complete histograms with both parts of the fitted curves with different vertical offsets for different age classes (2% between successive groups). The age class of 10–20 years is shown at the top, and the age class of 70–80 years is shown at the bottom. Whole ADC range (a) and reduced ADC range to visualize the step between the fitted curves of hist1 and hist2 (b).

The results of the fitting of the second part of the histogram using and the deviations are shown in Supplementary Table 3. The comparison of the second part of the mean histograms for the seven age classes with the fitted curves is shown in Figure 7(a). The fitted curves consist of three different components: the biexponential decay, a Gaussian curve to describe the local maximum at 2.8 · 10^−3^ mm^2^/s, and a straight line with a negative slope and a foot point at 4.3 · 10^−3^ mm^2^/s. These components are shown in Figure 7(b). The variation of the fitted parameters of the second part of the histogram for the different age classes is shown in Figure 8 again for all datasets in bold and in addition for the three subsets. The evaluation for male and female separately (Figure 9) showed no clear difference between the evaluated parameters. The comparison of the complete histograms with both parts of the fitted curves is shown in Figure 10(a). The small step between the fitted curves of hist1 and hist2 can be seen in Figure 10(b).

## 4. Discussion

This study confirms the strong dependency of the whole brain ADC histograms on the age of the examined subjects. Any comparison between different groups of subjects should therefore consider this influence. On the other hand, the shape of the mean whole brain ADC histograms of the different age classes can be described by the same model using different parameters. The model used in this paper has the same main components as that of previous studies: a brain tissue part, a pure CSF part, and a part describing the partial volume between the brain tissue and CSF. In the first approach, the brain tissue part was approximated by a single Gaussian function, as it was suggested by previous studies [17, 18]. In this case, the residuum of the ADC histograms of all age classes had a very similar shape (Figure 3(b)), indicating that the first part of the histogram is described incompletely. The position of the peak of the Gaussian function is changing in a very similar way as it was described by Watanabe et al. [19] (Figure 5(b)) and the increase of the width of the fitted curve with age was also found as described by Watanabe et al. In addition to their work, we also analyzed the age-dependent amplitudes of the Gaussian curves that were used to approximate the evaluated histograms.

The residuum could be considerably reduced in all age classes by the introduction of a combination of two Gaussian curves for the first part of the histogram (Figure 4(a)). In this case, both peaks showed a U-shaped behaviour in their age dependency (Figure 5(b)) with an almost constant difference for all ages of approximately 0.04 · 10^−3^ mm^2^/s. This difference is similar to the difference of the mean ADC values of white and gray matter in a recent study of Baumann et al. [21]. They found mean values of 0.713 · 10^−3^ mm^2^/s for white matter and 0.743 · 10^−3^ mm^2^/s for parietal gray matter (difference 0.030 · 10^−3^ mm^2^/s). The Gaussian curve with the lower peak position can therefore be assumed to be representative of white matter. This assumption is confirmed by the age dependence of the peak heights (Figure 6(a)): the peak of the first Gaussian curve is almost constant while the second curve decreases from 6.5% to 5% in the age range from 15 to 75 years. These results correspond to a decreasing relative gray matter volume and slightly increasing relative white matter volume over lifespan observed by Ziegler et al. [22] (Supplementary Material, Figure S2) and by Hasan et al. [23].

The second part of the whole brain histogram consists of a decreasing component and a Gaussian shape with a peak at about 2.8 · 10^−3^ mm^2^/s. The position of this Gaussian shape was similar to the CSF component in the work of Dyke et al. [17]. However, the decreasing part could not be described by an additional Gaussian curve; instead, we used a combination of a biexponential decay and a decreasing straight line. The foot point of the straight line was at 4.3 · 10^−3^ mm^2^/s. This value is much larger as the peak of the CSF Gaussian curve and might represent the apparent diffusion coefficient of moving CSF. The decreasing straight line is characterized by its value at the position 0.875 · 10^−3^ mm^2^/s, which shows a linear increase with age (Figure 8(a)). The Gaussian curve, representing the pixels of mainly CSF, has an amplitude that is increasing linearly up to the age of 45 years. With higher ages, the increase is larger. The shift of the peak of the Gaussian curve to larger ADC values with age might also be an effect of the changing moving characteristics of CSF molecules.

The amplitudes of both exponential decay curves are decreasing with age. The curves represent voxel with partial volume of CSF and brain tissue and the decrease of their amplitude corresponds to the decrease of the brain tissue components in the first part of the histogram. The similar shape of the residuum, the difference between the calculated histograms, and the fitted curve in Figure 6(a) for all age classes is a hint that this modeling of the second part of the whole brain histogram is still incomplete. However, for all age classes, the amplitude of the residuum is very small (it is magnified by a factor of three in Figure 6(a)).

With the proposed model for the whole brain ADC histogram, it was possible to fit the histograms of seven age classes and to demonstrate systematic changes of the histogram with age. This model can be used to characterize changes of the whole brain ADC histogram in certain diseases. The sensitivity for such changes was already shown by previous studies [6–8]. The differentiation of a normal and a pathologically changed ADC histogram needs a consideration of the subject's age and can be improved by using the proposed ADC histogram model. An ADC histogram characterization by this model may replace the use of derived parameters as percentiles and skewness of the ADC histogram.

## Supplementary Material

The averaged ADC histograms (for the whole patient group and for women and men separately) were splitted into part1 and a part2. The results of the fitting procedures of part1 due to model 1 with one Gaussian curve and model 2 with two Gaussian curves (equation 1 in the manuscript) for the different age classes are given in Tab. 1 and Tab. 2.
The results of the fitting procedures of part3 due to the model described in equation for the different age classes are given in Tab. 3.
