# Supplementary material for: Parameterization of the Age-Dependent Whole Brain Apparent Diffusion Coefficient Histogram
Source: Biomed Res Int. 2015 Nov 2;2015:373716. doi: 10.1155/2015/373716 (PMC4644831; doi:10.1155/2015/373716)
Supplement: Supplementary file 1 — The averaged ADC histograms (for the whole patient group and for women and men separately) were splitted into part1 and a part2. The results of the fitting procedures of part1 due to model 1 with one Gaussian curve and model 2 with two Gaussian curves (equation 1 in the manuscript) for the different age classes are given in Tab. 1 and Tab. 2. The results of the fitting procedures of part3 due to the model described in equation for the different age classes are given in Tab. 3. [file 373716.f1.pdf]

Tab.1: Results, root mean square deviation and relative deviation from the fitting of the first part of the histogram, hist1, model 1, whole group

Age Class	A [%]	μ [10^{-3} mm^2/s]	σ [10^{-3} mm^2/s]	rmsd [10^{-3} %]	relative rmsd [10^{-2}]
1	7.16	0.76	0.98	0.58	3.12
2	6.93	0.76	0.98	0.76	4.15
3	6.76	0.75	0.99	0.74	4.11
4	6.48	0.75	1.03	0.75	4.37
5	6.28	0.75	1.07	0.65	3.96
6	5.90	0.76	1.14	0.57	3.82
7	5.45	0.77	1.17	0.46	3.48

Tab.2a: Results from the fitting of the first part of the histogram, hist1, model 2, whole group

Age Class	A1 [%]	μ1 [10^{-3} mm^2/s]	σ1 [10^{-3} mm^2/s]	A2 [%]	μ2 [10^{-3} mm^2/s]	σ2 [10^{-3} mm^2/s]
1	6.48	0.77	1.08	0.92	0.74	0.62
2	6.18	0.77	1.10	0.99	0.73	0.60
3	5.92	0.76	1.12	1.08	0.73	0.63
4	5.72	0.76	1.16	1.00	0.72	0.65
5	5.45	0.76	1.24	1.13	0.73	0.72
7	4.89	0.78	1.34	0.84	0.75	0.76

Tab.2b: Root mean square deviation and relative deviation from the fitting of the first part of the histogram, hist1, model 2, whole group

Age Class	rmsd [10^{-3} %]	relative rmsd [10^{-2}]
1	0.26	1.37
2	0.25	1.36
3	0.24	1.37
4	0.26	1.52
5	0.23	1.44
6	0.22	1.50
7 0	.26	1.95

Tab.2c: Results from the fitting of the first part of the histogram, hist1, model 2, 3 subgroups

Subgroup	Age Class	A1 [%]	μ1 [10^{-3} mm^2/s]	σ1 [10^{-3} mm^2/s]	A2 [%]	μ2 [10^{-3} mm^2/s]	σ2 [10^{-3} mm^2/s]
1	1	6.42	0.77	1.09	0.95	0.74	0.61
1	2	6.25	0.76	1.05	0.88	0.72	0.57
1	3	5.88	0.76	1.13	1.09	0.72	0.64
1	4	5.71	0.76	1.15	0.99	0.72	0.63
1	5	5.50	0.77	1.25	1.09	0.73	0.70
1	6	5.29	0.78	1.31	1.05	0.73	0.70
1	7	4.75	0.79	1.33	0.94	0.74	0.81
2	1	6.54	0.77	1.08	0.89	0.75	0.62
2	2	6.24	0.76	1.09	0.94	0.73	0.61
2	3	5.94	0.77	1.12	1.07	0.73	0.63
2	4	5.77	0.77	1.16	0.93	0.73	0.64
2	5	5.53	0.76	1.23	1.08	0.72	0.68
2	6	5.16	0.78	1.37	1.00	0.73	0.73
2	7	4.71	0.79	1.37	0.97	0.74	0.80
3	1	6.42	0.77	1.07	0.91	0.74	0.64
3	2	6.17	0.77	1.11	0.96	0.74	0.62
3	3	5.99	0.76	1.10	1.07	0.72	0.62
3	4	5.79	0.76	1.16	0.99	0.72	0.64
3	5	5.46	0.77	1.24	1.10	0.72	0.68
3	6	5.23	0.78	1.32	1.04	0.73	0.71
3	7	4.80	0.78	1.31	0.97	0.74	0.80

Tab.2d: Root mean square deviation and relative deviation from the fitting of the first part of the histogram, hist1, model 2, 3 subgroups

Subgroup	Age Class	rmsd [10^{-3} %]	relative rmsd [10^{-2}]
1	1	0.28	1.52
1	2	0.29	1.56
1	3	0.24	1.35
1	4	0.31	1.79
1	5	0.23	1.40
1	6	0.22	1.45
1	7	0.31	2.34
2	1	0.25	1.33
2	2	0.24	1.30
2	3	0.26	1.47
2	4	0.25	1.46
2	5	0.22	1.35
2	6	0.21	1.44
2	7	0.23	1.81
3	1	0.27	1.47
3	2	0.31	1.71
3	3	0.28	1.54
3	4	0.23	1.31
3	5	0.22	1.37
3	6	0.23	1.54
3	7	0.27	2.00

Tab.2e: Results from the fitting of the first part of the histogram, hist1, model 2, male and female patients

Sex	Age Class	A1 [%]	μ1 [10^{-3} mm^2/s]	σ1 [10^{-3} mm^2/s]	A2 [%]	μ2 [10^{-3} mm^2/s]	σ2 [10^{-3} mm^2/s]
female	1	6.46	0.77	1.09	0.89	0.73	0.61
female	2	6.22	0.76	1.08	0.97	0.73	0.60
female	3	5.94	0.76	1.10	1.13	0.73	0.63
female	4	5.73	0.76	1.15	1.01	0.72	0.63
female	5	5.51	0.77	1.23	1.14	0.72	0.67
female	6	5.27	0.78	1.29	1.10	0.73	0.71
female	7	4.69	0.78	1.28	1.01	0.74	0.83
male	1	6.52	0.77	1.07	0.89	0.75	0.61
male	2	6.12	0.77	1.11	0.97	0.73	0.61
male	3	5.96	0.77	1.15	0.97	0.73	0.63
male	4	5.79	0.76	1.18	0.95	0.73	0.64
male	5	5.46	0.77	1.26	1.09	0.72	0.70
male	6	5.11	0.78	1.36	1.06	0.73	0.74
male	7	4.57	0.79	1.38	1.05	0.75	0.81

Tab.2f: Root mean square deviation and relative deviation from the fitting of the first part of the histogram, hist1, model 2, male and female patients

Sex	Age Class	rmsd [10^{-3} %]	relative rmsd [10^{-2}]
female	1	0.28	1.51
female	2	0.30	1.65
female	3	0.24	1.33
female	4	0.24	1.39
female	5	0.25	1.50
female	6	0.23	1.50
female	7	0.39	2.88
male	1	0.24	1.31
male	2	0.25	1.41
male	3	0.24	1.40
male	4	0.25	1.46
male	5	0.22	1.36
male	6	0.20	1.38
male	7	0.22	1.74

Tab3a: Results from the fitting of the second part of the histogram, hist2, all patients

Age Class	A3 [%]	k3 [10^{-3} mm^2/s]	A4 [%]	k4 [10^{-3} mm^2/s]	A5 [10^3 s/mm^2]	μ5 [%]	A6 [%]	μ6 [10^{-3} mm^2/s]	σ6 [10^{-3} mm^2/s]
1	3.82	0.07	1.46	0.28	0.06	0.21	0.05	2.49	2.20
2	3.36	0.07	1.26	0.31	0.07	0.24	0.09	2.64	2.29
3	3.24	0.07	1.08	0.32	0.08	0.27	0.18	2.69	2.67
4	2.92	0.07	1.03	0.32	0.09	0.31	0.24	2.70	2.82
5	2.99	0.08	0.86	0.34	0.10	0.33	0.34	2.71	2.69
6	3.08	0.09	0.67	0.43	0.11	0.37	0.45	2.74	2.91
7	3.16	0.10	0.51	0.49	0.12	0.42	0.64	2.79	2.94

Tab3b: Root mean square deviation and relative deviation from the fitting of the second part of the histogram, hist2, all patients

Age Class	rmsd [10^{-3} %]	relative rmsd [10^{-2}]
1	0.03	1.39
2	0.02	1.06
3	0.02	0.86
4	0.02	0.88
5	0.03	1.05
6	0.03	1.13
7	0.04	1.36

Tab.3c: Results from the fitting of the second part of the histogram, hist2, 3 subgroups

Sub Group	Age Class	A3 [%]	k3 [10^{-3} mm^2/s]	A4 [%]	k4 [10^{-3} mm^2/s]	A5 [10^{3} s/mm^2]	μ5 [%]	A6 [%]	μ6 [10^{-3} mm^2/s]	σ6 [10^{-3} mm^2/s]
1	1	3.62	0.07	1.54	0.26	0.06	0.22	0.06	2.50	1.80
1	2	3.20	0.06	1.25	0.31	0.07	0.24	0.09	2.64	2.15
1	3	3.06	0.07	1.12	0.31	0.08	0.28	0.17	2.68	2.58
1	4	2.70	0.07	1.07	0.30	0.09	0.32	0.22	2.69	2.85
1	5	3.14	0.08	0.79	0.36	0.10	0.33	0.36	2.69	2.56
1	6	3.16	0.09	0.68	0.41	0.10	0.36	0.44	2.74	2.96
1	7	3.09	0.10	0.55	0.51	0.12	0.40	0.66	2.78	3.10
2	1	4.06	0.07	1.48	0.28	0.06	0.20	0.04	2.43	2.69
2	2	3.48	0.07	1.19	0.31	0.07	0.24	0.10	2.62	2.21
2	3	3.46	0.07	1.01	0.32	0.08	0.28	0.18	2.69	2.57
2	4	3.10	0.07	1.03	0.33	0.09	0.30	0.24	2.70	2.79
2	5	3.00	0.08	0.85	0.36	0.09	0.32	0.32	2.72	2.74
2	6	3.02	0.10	0.66	0.44	0.11	0.39	0.46	2.74	2.88
2	7	3.26	0.11	0.45	0.53	0.12	0.42	0.70	2.81	2.90
3	1	3.74	0.07	1.40	0.30	0.06	0.21	0.05	2.54	2.48
3	2	3.39	0.07	1.33	0.30	0.07	0.25	0.09	2.66	2.43
3	3	3.25	0.07	1.08	0.32	0.07	0.26	0.19	2.68	2.92
3	4	2.97	0.07	1.01	0.32	0.09	0.31	0.24	2.72	2.78
3	5	2.90	0.08	0.90	0.32	0.10	0.35	0.34	2.73	2.70
3	6	3.06	0.09	0.67	0.42	0.11	0.38	0.43	2.75	2.86
3	7	3.24	0.10	0.48	0.63	0.11	0.37	0.64	2.80	3.00

Tab.3d: Root mean square deviation and relative deviation from the fitting of the second part of the histogram, hist2, 3 subgroups

Subgroup	Age Class	rmsd [10^{-3} %]	relative rmsd [10^{-2}]
1	1	0.03	1.58
1	2	0.03	1.21
1	3	0.02	0.78
1	4	0.02	0.92
1	5	0.04	1.49
1	6	0.04	1.38
1	7	0.05	1.48
2	1	0.04	1.74
2	2	0.02	1.05
2	3	0.03	1.29
2	4	0.03	1.05
2	5	0.03	1.17
2	6	0.03	1.09
2	7	0.06	1.70
3	1	0.03	1.35
3	2	0.03	1.54
3	3	0.02	1.02
3	4	0.03	1.04
3	5	0.03	0.98
3	6	0.03	1.21
3	7	0.05	1.48

Tab.3e: Results from the fitting of the second part of the histogram, hist2, male and female patients

Sex	Age Class	A3 [%]	k3 [10^{-3} mm^2/s]	A4 [%]	k4 [10^{-3} mm^2/s]	A5 [10^3 s/mm^2]	μ5 [%]	A6 [%]	μ6 [10^{-3} mm^2/s]	σ6 [10^{-3} mm^2/s]
female	1	3.65	0.07	1.45	0.30	0.06	0.20	0.04	2.52	2.52
female	2	3.35	0.07	1.25	0.32	0.07	0.23	0.09	2.62	2.10
female	3	3.13	0.07	1.13	0.31	0.08	0.26	0.17	2.70	2.65
female	4	2.84	0.07	1.03	0.32	0.09	0.31	0.23	2.72	2.75
female	5	2.98	0.08	0.86	0.36	0.09	0.32	0.33	2.71	2.61
female	6	3.19	0.09	0.71	0.44	0.10	0.34	0.41	2.75	2.86
female	7	3.10	0.10	0.58	0.44	0.12	0.41	0.56	2.82	2.86
male	1	3.91	0.07	1.49	0.27	0.06	0.22	0.05	2.48	2.00
male	2	3.36	0.07	1.28	0.29	0.08	0.26	0.11	2.67	2.60
male	3	3.37	0.07	1.02	0.33	0.08	0.28	0.20	2.65	2.91
male	4	3.05	0.08	1.02	0.32	0.09	0.31	0.24	2.68	2.87
male	5	2.96	0.08	0.93	0.31	0.10	0.36	0.34	2.72	2.72
male	6	3.01	0.09	0.66	0.41	0.12	0.40	0.47	2.73	2.93
male	7	3.26	0.11	0.44	0.76	0.11	0.37	0.76	2.77	3.03

Tab.3f: Mean square deviation and relative deviation from the fitting of the second part of the histogram, hist2, male and female patients

Sex	Age Class	rmsd [10^{-3} %]	relative rmsd [10^{-2}]
female	1	0.03	1.39
female	2	0.02	1.05
female	3	0.02	1.05
female	4	0.02	0.80
female	5	0.03	1.25
female	6	0.04	1.28
female	7	0.05	1.57
male	1	0.03	1.53
male	2	0.03	1.35
male	3	0.03	1.10
male	4	0.03	1.12
male	5	0.03	1.08
male	6	0.03	1.12
male	7	0.05	1.42
